# Serious bronchospasm induced by cisatracurium besylate: A case report

**DOI:** 10.1097/MD.0000000000025516

**Published:** 2021-04-16

**Authors:** Ning Wang, Yaozhong Zhang, Yu Hu, Qiyao Yang, Zhenbo Su

**Affiliations:** Department of Anesthesiology, China-Japan Union Hospital of Jilin University, Changchun, China.

**Keywords:** anaphylactoid reaction, anesthesia, bronchospasm, cis-atracurium

## Abstract

**Rationale:**

Cis-atracurium as an intermediate-acting non-depolarizing neuromuscular blocker is widely used clinically with less causing cyclic fluctuations and less histamine release. As the use rate increases, allergic reactions and anaphylactoid reactions caused by cis-atracurium increase.

**Patient concerns:**

A 23-year-old woman underwent laparoscopic bariatric surgery. Airway spasm occurred after anesthesia induction and the operation was suspended. After adjustment, the anesthesia was performed with the same anesthetic scheme again. After induction, skin flushing and airway resistance increased, then the symptoms were relieved. When the cis-atracurium was given again, the symptoms of airway spasm reappeared immediately, and after communicating with the family, the operation was successfully completed with rocuronium.

**Diagnoses:**

Serious bronchospasm induced by cisatracurium besylate.

**Interventions:**

The patient was undergone assisted ventilation with continuous positive airway pressure (CPAP) and aminophylline 250 mg, methylprednisolone 80 mg were given intravenously.

**Outcomes:**

There was no any obvious discomfort in the patient's self-report during the next day's visit. The patient was discharged 7 days later. No abnormalities were observed during following 4 weeks.

**Lessons:**

Although the anaphylactoid reactions caused by cis-atracurium are rare, the bronchospasm and anaphylactic shock caused by it greatly increase the risk of anesthesia, which should be taken seriously by clinicians. Increased vigilance in diagnosis, and treatment are essential to prevent aggravation and further complication.

## 1 Introduction

Allergic reactions caused by anesthetic drugs are very rare. Allergens that are more common during perioperative period are allergic to artificial colloids, antibiotics, and implants. A 2-year study in France (January 1, 1997–December 31, 1998) showed that the most common allergen causing anaphylaxis during anesthesia was neuromuscular blockers (69.2%) and latex (12.1%).^[1]^ The 6th National Audit Project (NAP6) on perioperative anaphylaxis in Britain showed that antibiotics were the main allergens causing perioperative anaphylaxis, accounting for 48.9%, followed by neuromuscular blockers, accounting for 33.8%.^[2]^ Succinylcholine (60.6%) was the most frequently incriminated NMBA, whereas the low frequency of reactions involving cis-atracurium was confirmed (5.9%) when market shares of each NMBA were taken into account.^[3]^ We report a case of an anaphylactoid reaction following induction of anesthesia with cis-atracurium. We introduced the mechanism of anaphylactic reaction and anaphylactoid reaction in detail, with how to diagnose them.

## 2 Case presentation

The patient, woman, 23 years old, height 170 cm, weight 137 kg, body mass index (BMI) 47.4 kg/m^2^, entered our hospital on November 7, 2018 to perform laparoscopic bariatric surgery under general anesthesia. She was evaluated class III risk using the American Society of Anesthesiologists (ASA) criteria and did not have respiratory, circulatory diseases, liver, kidney, or other organ dysfunction. She had a history of penicillin allergy, appendectomy under local anesthesia, and cesarean section under spinal anesthesia. She has smoked for 7 years, 7 cigarettes/d, and has stopped smoking for 4 days before the operation. With preoperative prohibition of drinking for 6 hours, fasting for 12 hours, she has been monitored routinely and has been measured radial artery pressure when entered the operating room at 8:30 am on November 12, 2018. We provided the patient with 8 L/min high-flow mask oxygen. The arterial blood pressure (ABP) was 158/76 mm Hg. The heart rate (HR) was 78 beats/min. The peripheral oxygen saturation (SpO_2_) measured using pulse oximetry was 99%. The results of arterial blood gas analysis were as follows: arterial partial pressure of carbon dioxide (PaCO_2_), 44.3 mm Hg, arterial partial pressure of oxygen (PaO_2_), 260 mm Hg; and arterial oxygen saturation (SaO_2_) 100%. After 10 minutes of oxygen inhalation, anesthesia was induced with intravenous (IV) penehyclidine 1 mg (Chengdu Lisite Pharmaceutical Co., Ltd., Jinjiang Industrial Development Zone, Chengdu, H200220606, 180703), prednisolone 20 mg (Jiangxi Sinopharm Co., Ltd., No.888, Guoyao Avenue, Xiaolan Industrial Park, Nanchang City, Jiangxi Province, H36022365, 19031022), midazolam 10 mg (Jiangsu Enhua Pharmaceutical Co., Ltd., No.18, Yangshan Road, Xuzhou Economic Development Zone, H10980025, 20181203), propofol 150 mg (Sichuan Guorui Pharmaceutical Co., Ltd., North section of Fenghuang Road, Yujin Town, Qianwei County, Leshan City, H200330115, 1905161), cis-atracurium 40 mg (Jiangsu Hengrui Pharmaceutical Co., Ltd., No. 38 the Yellow River Road, Lianyungang economic and Technological Development Zone, H20060869 , 18072721), and Sufentanil 20 μg (Yichang Renfu Pharmaceutical Co., Ltd., No. 19 Dalian Road, Yichang Development Zone, Hubei, H20054171, 91A03051A1). After tracheal intubation, breathing sounds were not heard in both lungs, and end tidal CO_2_ (ETCO_2_) can be seen continuously waveform. Auscultating again, we could hear the weak breath of both lungs, and a lot of dry and wet rales, then we used negative pressure to suck sputum with only a small amount of clear secretions. A lot of dry and wet rales could still been heard at this time. With hand-controlled ventilation, airway pressure (Paw) was 40 cmH_2_O, and tidal volume (*V*_t_) was 350 mL. At this point PaCO_2_ was 50.6 mmHg, PaO_2_ 81 mm Hg, SaO_2_ was 95%, SpO_2_ was 95%, and fraction inspired O_2_(FiO_2_) was 90%. We considered bronchospasm, followed by intravenous injection of aminophylline 250 mg, methylprednisolone 80 mg. After 15 minutes, the results of arterial blood gas analysis were as follows: PaCO_2_ was 46.3 mm Hg, PaO_2_ was 80 mm Hg, SaO_2_ was 95%, SpO_2_ was 95%, and FiO_2_ was 90%. Hemodynamics showed no significant fluctuation and respiratory compliance improved. After 10 minutes, PaCO_2_ was 40.6 mm Hg, PaO_2_ was 103 mm Hg, SaO_2_ was 98%, SpO_2_ was 96%, and FiO_2_ was 90%. Pressure controlled ventilation was used after intubation, Paw was 38 cmH_2_O, *V*_t_ was 350 mL, respiratory rate (RR) was 12 breath/min, positive end-expiratory pressure (PEEP) was 5 cmH_2_O, and slope was 1.0 seconds. Please consult the Department of Respiratory Medicine. The consultation opinion indicates that the patient has a long history of smoking, and the airway sensitivity is likely to cause airway spasm after endotracheal intubation. We informed the patient's family related risks fully and obtained the consent of the patient's family to suspend the operation. After 1 hour, Paw was 35 cmH_2_O, *V*_t_ was 450 mL, RR was 12 breath/min, PEEP was 5 cmH_2_O, slope was 1.0 seconds, and respiratory compliance was significantly improved. Return to intensive care unit safely under moderate sedation. Respiratory function recovered completely after 2 hours. We removed the tracheal tube. At this time, PaCO_2_ was 40.1 mmHg, PaO_2_ was 96 mm Hg, SaO_2_ was 98%, SpO_2_ was 96%, FiO_2_ was 40%. The next day, the patient reported tracheal burning sensation, which was considered by the pressure injury of the airway. After preparation by atomization and oxygen therapy, the operation was performed again on November 21, 2018. Intramuscular injection of atropine 1 mg, intravenous drip of aminophylline 250 mg, and methylprednisolone 80 mg to prevent airway spasm. At this time, PaCO_2_ was 44.2 mmHg, PaO_2_ was 243 mmHg, SaO_2_ was 100%, SpO_2_ was 99%, FiO_2_ was 50%. The drug and dose for anesthesia induction were the same as the first time, and the Hand-controlled ventilation was given after the spontaneous breathing disappears. During ventilation, airway resistance was high, skin of anterior chest and neck was flushed, and there was no obvious hemodynamic fluctuation. After tracheal intubation, anesthesia was maintained with sevoflurane in 50% oxygen in air. A large number of dry and wet rales could been heard in both lungs during auscultation. The airway resistance increased significantly. After 5 minutes, the symptoms of skin flushing gradually disappeared. At this moment, PaCO_2_ was 41.6 mmHg, PaO_2_ was 98 mmHg, SaO_2_ was 98%, SPO_2_ was 98%, FiO_2_ was 90%. Then, the pressure ventilation mode was given, *P*_plat_ was 28 cmH_2_O, RR was 12 breath/min, PEEP was 7 cmH_2_O, slope was 1.0 seconds, *V*_t_ was 522 mL. After 1 hour, the rales gradually disappeared and the breath sound was clear. Re-examination of arterial blood gas analysis were as follows: PaCO_2_ was 49.5 mm Hg, PaO_2_ was 135 mm Hg, SaO_2_ was 99%, SpO_2_ was 99%, FiO_2_ was 90%. At this time, the patient's breathing gradually recovered, and 5 mg of cis-atracurium was given again. The airway pressure increased immediately. Auscultation of the lungs again showed a large amount of dry rales, considering anaphylactoid reaction caused by cis-atracurium which induced the bronchial spasm. Respiratory consultation pointed out that under general anesthesia tracheal intubation, the left lung breath sounds weak, the right lower lung was covered with biphasic high-pitched dry sounds. Symptoms such as skin flushing and bronchospasm appeared immediately after induction of this patient, and cis-atracurium was considered to cause anaphylactoid reaction. When the symptoms were gradually relieved, we gave cisatracurium again with bronchospasm symptoms immediately developed, while bronchospasm did not occur again when rocuronium was given. It was equivalent to carry out drug provocation test without knowing it. Drug provocation test is the golden standard for finding allergens, so it was determined that the bronchospasm was caused by cis-atracurium.

Considering airway sensitivity, airway spasm (not excluding drug allergy) under high-sensitivity state was at high risk of operation. After actively communicating with the patient's family and explaining the associated risks, the patient's family indicated that the risk was approved for surgery. Switch to the muscle relaxant rocuronium bromide (Eccorson) 20 mg (N.V.ORGANON, Molenstraat 110 P.O.BOX 20 OSS 5340 BH Netherlands, H20140847, R006942), no other allergic reactions such as skin flushing. After that, surgery was started. During the operation, rocuronium 20 to 30 mg/h and sufentanil 5 to 10 μg/h were added on time, and a total of 2000 mL Ringer lactated solution was infused. No allergic reaction occurred, no obvious hemodynamic fluctuation was observed, and airway resistance gradually reduced. After 4 hours, the operation was completed. At this time, PaCO_2_ was 56.2 mmHg, PaO_2_ was 138 mmHg, SaO_2_ was 99%, SpO_2_ was 99%, FiO_2_ was 90%. Methylprednisolone 80 mg and sufentanil 50 μg were given again for the transition of respiratory function recovery. After the operation, the patient returned to the intensive care unit. After 2 hours, the tube was removed and the results of arterial blood gas analysis were as follows: PaCO_2_ was 52.1 mm Hg, PaO_2_ was 103 mm Hg, SaO_2_ was 97%, SpO_2_ was 98%, FiO_2_ was 30%. There was no any obvious discomfort in the patient's self-report during the next day's visit, rales completely disappeared. The patient was discharged 7 days later. No abnormalities were observed during following 4 weeks.

## 3 Discussion

The clinical manifestations of perioperative allergic reactions and anaphylactoid reactions are related to the amount of histamine releasing from mast cells, usually involving multiple systems and multiple organs, which can be life-threatening in severe cases.^[4]^ Hypersensitivity reactions occurring during anesthesia remain a major cause of concern for anesthesiologists. Neuromuscular blockers are the major allergens that cause allergic reactions and anaphylactoid reactions during anesthesia. Cis-atracurium can cause an allergic reaction as an antigen, and can also cause an anaphylactoid reaction by histamine release. The patient was not exposed to latex and antibiotics before the allergy occurred, so latex and antibiotic allergies were not considered. Since it was an allergic reaction that occurrence after induction, it was necessary to mainly consider the allergy of the inducing drug. The patient had undergone painless gastroscopy which used propofol and sufentanil before admission. During the operation, propofol and sufentanil were added regularly. After the operation, 50 μg of sufentanil was given again for transitional extubation. No allergic reactions occurred, and it was unlikely that allergic reactions to propofol and sufentanil are considered. Midazolam is a safe drug because it does not produce active metabolites that are thought to cause cross-allergic reactions with other benzodiazepines. A total of 2 cases of allergic reactions caused by intravenous injection have been reported worldwide,^[5,6]^ an anaphylactoid reaction caused by intravenous injection,^[7]^ 1 case of allergic reaction caused by intramuscular injection.^[8]^ There are no reports of allergies in penehyclidine and prednisolone.

Perioperative allergic reactions are type I allergic reactions. Allergens first enter the body to produce high levels of immunoglobulin E through T cells and B cells, and then bind to mast cells and basophils surface receptors to make the body sensitized. When the body contacts the same allergen again, it will produce degranulation of mast cells and basophils, and rapid release of a large amount of histamine, bradykinin, prostaglandin, and other active media causes a series of clinical symptoms. Clinically applied neuromuscular blockers have quaternary ammonium groups, and some foods, drugs, and cosmetics also contain similar groups.^[9]^ When entering the human body, it can presensitize mast cells and basophils. When cis-atracurium enters the body, allergic reactions can occur and there is a risk of cross-allergy. According to reports,^[10]^ incidence of allergic reactions are as follows: Rocuronium 8.0/100,000, vecuronium 2.8 /100,000, atracurium 4.01 /100,000; and in patients with Rocuronium allergy, the incidence of cross-allergy to other muscle relaxants is: succinylcholine (44%) and vecuronium (40%), while pancuronium 19% and atracurium 20%, cisatracurium 5%. Therefore, when a neuromuscular blocker is allergic, be careful to use other types of neuromuscular blockers or alternative anesthesia without nerve blockers.^[11]^ Goikoetxea et al^[12]^ have also reported that anaphylactic shock had occurrence immediately after induction with cis-atracurium in patients underwent radical resection of rectal cancer under general anesthesia. One week later, skin tests and basophilic activated granulocyte experiments confirmed that the allergic reaction was caused by cis-atracurium. Recent studies have shown that hairdressers who are frequently exposed to quaternary ammonium ion compounds have a significant increase in allergic reactions to neuromuscular blockers, so repeated exposure to quaternary ammonium-containing compounds is also one of the sensitizing risk factors for neuromuscular blockers.^[13]^

Most of the perioperative anaphylactoid reactions induced by neuromuscular blockers are caused by non-immune mechanisms that directly release the medium from mast cells and basophils, or directly activate complements, leading to systemic histamine release. Clinical symptoms are similar to anaphylactic reactions. Recent studies by Che et al^[14]^ have shown that cis-atracurium triggers LAD2 cell degranulation and release of inflammatory precursors through G protein-coupled receptor MRGPRX2 to produce anaphylactoid reactions. It is known that most neuromuscular blockers cause non-specific histamine release from mast cells, and benzylisoquinolines are more likely to cause histamine release than aminoguanidines.^[15]^ If anaphylactoid reactions occurs, we can use a neuromuscular blocker with a low rate of histamine release to replace the neuromuscular blocker with a higher rate of histamine release. In this case, no anaphylactoid reactions occurred after replacing the benzylisoquinoline cis-atracurium with aminoguanidine rocuronium. It was confirmed from the side that anaphylactoid reactions occurred in this case rather than allergic reactions.

Treatment and diagnosis of allergic reaction: when suspected allergic reactions in patients, all suspected sensitizing drugs should be stopped immediately, anti-allergy treatment should be carried out. Meanwhile, blood samples should be taken for serum tryptase detection, because serum tryptase increases with the activation of mast cells, which is the only useful blood test during an acute allergic reaction. Although it can confirm the occurrence of anaphylaxis, it is not helpful for the detection of allergens. In order to determine whether an immune-mediated allergic reaction occurs, a prick test can be performed 4 to 6 weeks after the occurrence of allergic reaction. Because a large amount of inflammatory factors such as histamine will be consumed in the event of an allergic reaction, a false negative result may occur in an immediate prick test. However, some scholars believe that it is meaningful to do the prick experiment immediately. The intradermal test was performed when the prick test was negative. All suspected drugs should be tested during prick test and intradermal test, and all drugs of the same type should be tested to prevent cross-allergic reactions between drugs, and non-allergic drugs can be selected for emergencies. Drug provocation test is the golden standard for finding allergens, but drug provocation test is risky and generally not recommended.^[16]^ No matter what kind of test is carried out, it should be carried out in the professional allergy and immunity department, and adrenaline and other rescue drugs should be prepared to avoid unforeseen events.

Diagnosis of anaphylactoid reactions: The diagnosis of anaphylactoid reactions is difficult, and there is no good laboratory diagnostic method. At present, there is no recognized standard model of anaphylactoid detection at home and abroad. Anaphylactoid reactions are generally an exclusive diagnosis. Histamine, β-hexosaminidase, and tryptase are common biomarkers for anaphylactoid reactions and anaphylactic reactions. When anaphylaxis occurs, the specific immunoglobulin E increases correspondingly except for the above biomarkers, while the level of immunoglobulin E does not increase in anaphylactoid reaction. Inquiry about medical history: There is a sensitization process in the history of anaphylactic reactions, whereas anaphylactoid reactions have no sensitization process.

It is difficult to distinguish allergic reactions from anaphylactoid reactions in clinic. Under uncertain circumstances, other types of neuromuscular blockers cannot be easily substituted for each other. In this case, we inferred that anaphylactoid reaction occurred in the patient by inquiring about the medical history, and no anaphylactoid reactions occurred in this patient who used aminosterol rocuronium instead of benzylisoquinoline cis-atracurium during the operation, which confirmed our diagnosis. However, blood samples were not preserved during the operation, and patients refused further allergen diagnostic tests after surgery. We still lack laboratory evidence.

## 4 Conclusion

In conclusion, although the anaphylactoid reactions caused by cis-atracurium are rare, the bronchospasm and anaphylactic shock caused by it greatly increase the risk of anesthesia, which should be taken seriously by clinicians. Once an allergic reaction or anaphylactoid reaction is suspected, the anesthesiologist should record the real-time relevant information in detail and immediately save the serum samples of the allergic person for subsequent testing and diagnosis. Increased vigilance in diagnosis, and treatment are essential to prevent aggravation and further complication.

## Author contributions

**Conceptualization:** Yaozhong Zhang.

**Data curation:** Qiyao Yang.

**Formal analysis:** Yu Hu.

**Investigation:** Yu Hu.

**Methodology:** Ning Wang.

**Project administration:** Yaozhong Zhang.

**Supervision:** Qiyao Yang.

**Validation:** Ning Wang.

**Writing – original draft:** Ning Wang.

**Writing – review & editing:** Zhenbo Su.
